# Assessing Prevalence and Factors Related to Frailty in Community-Dwelling Older Adults: A Multinomial Logistic Analysis

**DOI:** 10.3390/jcm10163576

**Published:** 2021-08-14

**Authors:** Encarnación Blanco-Reina, Lorena Aguilar-Cano, María Rosa García-Merino, Ricardo Ocaña-Riola, Jenifer Valdellós, Inmaculada Bellido-Estévez, Gabriel Ariza-Zafra

**Affiliations:** 1 Pharmacology and Therapeutics Department, Instituto de Investigación Biomédica de Málaga-IBIMA, School of Medicine, University of Málaga, 29016 Málaga, Spain; ibellido@uma.es; 2 Physical Medicine and Rehabilitation Department, Hospital Regional Universitario, 29010 Málaga, Spain; loagca2011@hotmail.com; 3 Health District of Málaga-Guadalhorce, 29009 Málaga, Spain; rosaballet@yahoo.es (M.R.G.-M.); jenny_dok7@hotmail.com (J.V.); 4 Escuela Andaluza de Salud Pública, 18011 Granada, Spain; ricardo.ocana.easp@juntadeandalucia.es; 5 Instituto de Investigación Biosanitaria ibs.GRANADA, 18011 Granada, Spain; 6 Geriatrics Department, Complejo Hospitalario Universitario, 02006 Albacete, Spain; gariza@sescam.jccm.es

**Keywords:** frailty, older adults, polymedication, osteoarticular pathology, psychopathology, primary care

## Abstract

Frailty is an age-related clinical condition that typically involves a deterioration in the physiological capacity of various organ systems and heightens the patient’s susceptibility to stressors. For this reason, one of the main research goals currently being addressed is that of characterising the impact of frailty in different settings. The main aim of this study is to determine the prevalence of Fried’s frailty phenotype among community-dwelling older people and to analyse the factors associated with frailty. In this research study, 582 persons aged 65 years or more participated in this cross-sectional study that was conducted at primary healthcare centres in Málaga, Spain. Sociodemographic, clinical, functional and comprehensive drug therapy data were compiled. The relationship between the independent variables and the different states of frailty was analysed by using a multinomial logistic regression model. Frailty was present in 24.1% of the study sample (95% CI = 20.7–27.6) of whom 54.3% were found to be pre-frail and 21.6% were non-frail. The study variable most strongly associated with frailty was the female gender (OR = 20.54, 95% CI = 9.10–46.3). Other factors found to be associated with the state of frailty included age, dependence for the instrumental activities of daily living (IADL), polymedication, osteoarticular pathology and psychopathology. This study confirms the high prevalence of frailty among community-dwelling older people. Frailty may be associated with many factors. Some of these associated factors may be preventable or modifiable and, thus, provide clinically relevant targets for intervention. This is particularly the case for depressive symptoms, the clinical control of osteoarthritis and the use of polypharmacy.

## 1. Introduction

Population ageing is a global phenomenon that is producing significant sociodemographic transformations. According to the 2015 EU Ageing Report, the age demographic of the European population will change dramatically over the coming decades, with older people accounting for an increasing proportion out of the total. By 2060, persons aged over 65 years are expected to account for 28% of the total population in Europe (currently 18%), while the proportion of individuals over 80s will increase from 5% to 12% during the same period [1]. However, there is little evidence that increased longevity is accompanied by an extended period of good health [2]. In this respect, the WHO has published a *World Report on Ageing and Health*, reviewing current knowledge, identifying gaps and providing a public health framework for action. This report redefines healthy ageing, focusing on the notion of functional ability. In this respect, the authors remark that comprehensive assessments of functioning in older age are much better predictors of survival and other outcomes than the presence/absence of disease or even the extent of comorbidities. According to this report, the foremost condition among geriatric syndromes producing a negative impact on survival is the condition of frailty [3].

Without question, frailty will be one of the most serious public health challenges facing the world during this century [4]. Frailty is an age-related clinical condition that typically involves a deterioration in the physiological capacity of various organ systems [5,6,7] and heightens the patient’s susceptibility to stressors [5,6,7,8,9,10]. When stressor events (such as acute illness) occur, the functional capacity of a person with frailty deteriorates rapidly. Frailty often precedes disability [5,8], although the two conditions may coexist [11]. Quite evidently, older persons with frailty are more likely to present unmet care needs, to suffer falls and/or fractures, to require hospitalisation, to have a reduced quality of life and to be subject to iatrogenic complications and early mortality [5,6,8,12,13]. Studies have shown that frailty is a dynamic entity that may be encountered on a continuum ranging from fit to frail. Moreover, an individual’s level of frailty is liable to change in either direction (i.e., worsening or improving) over time [4]. In fact, a substantial proportion of the population experience at least one such transition [14,15,16], and thus the process is potentially reversible. Frailty is not an inevitable consequence of ageing, and therefore a strong focus on early screening and diagnosis is needed to enable optimal prevention.

Increasing numbers of persons with frailty are attending their general practitioners. Primary care is the first point of contact for most such persons. Attention is usually comprehensive and personalised, and thus this healthcare environment is very suitable for the identification, management and study of frailty [17,18]. However, the absence of a consensus on how to define and measure frailty presents challenges for research and clinical practice. Among the many operational definitions of frailty that have been proposed, the best known are Fried’s Frailty Phenotype [5] and Rockwood’s Frailty Index [9]. The heterogeneity of assessment instruments is one of the factors underlying the wide range of prevalence estimates that have been reported for frailty (4–59%) [19].

Frailty is an important public health issue for several reasons: Firstly, it is highly prevalent and is the condition that most commonly results in death; secondly, the process is potentially preventable and treatable, particularly with early intervention; finally, despite its prevalence, frailty is either not recognised as a clinical or diagnostic syndrome, or (on many occasions) it is not recorded in clinical charts. Accordingly, one of the main research goals currently being addressed is that of characterising the impact of frailty on the population [20]. In this respect, the ADVANTAGE initiative “Joint Action on the Prevention of Frailty”, which is co-funded by the Third European Health Programme (2014–2020), has highlighted the need for studies to be undertaken in order to determine the current prevalence of frailty in different settings [21]. Moreover, due to the multidimensional nature of frailty, diverse factors may be involved in its progression. Given the real possibility that frailty may transition and/or be reversed, we consider it of interest to investigate the question of frailty among older adults living in Spain, where life expectancy is among the highest in the world [22]. In this study, we assess clinical, functional and pharmacological aspects of risk factors for frailty. We consider many variables, some of which are potentially modifiable by targeted interventions and preventive actions. Specifically, our study aim is to determine the prevalence of Fried’s frailty phenotype among community-dwelling older people and to analyse the factors associated with frailty.

## 2. Materials and Methods

### 2.1. Study Design, Setting and Participants

In this cross-sectional study, all participants met the following inclusion criteria: aged 65 years or more, registered in the database of the Spanish NHS and belonging to the outpatient setting (not institutionalised). All participants provided informed consent to take part in the study. The study population was composed of 89,615 persons living in the community in Malaga, Spain. Patients were recruited at twelve primary healthcare centres by using stratified random sampling designed to obtain a representative sample. The study population was allocated in proportion to the size of each centre. Based on a published prevalence of frailty in primary care of 30% [23,24,25] and assuming a 4% margin of error and a 95% confidence interval, we calculated that the minimum sample size required for this study would be 502 persons, a figure that was increased by 15% to offset possible losses.

### 2.2. Data Collection and Global Assessment

In order to obtain the study data for analysis, patients were interviewed using a structured questionnaire. Further data were obtained from medication packaging and digital medical records. The questionnaire was used to obtain detailed information on the patients’ regular drug use, together with clinical, functional and sociodemographic data. Clinical diagnoses were examined, and the Charlson Comorbidity Index (CCI) [26] was calculated. The Spanish version (Nestlé Nutrition Institute) of the Mini Nutritional Assessment Short Form (MNA) was used for nutritional screening [27]. The patients’ independence in performing instrumental activities of daily living (IADL) was assessed using the Lawton scale [28]. Cognitive function was evaluated by using the short portable mental state questionnaire by Pfeiffer (SPMSQ) [29], and mood status was determined by using the Geriatric Depression Scale (GDS-15) by Yesavage [30].

### 2.3. Measuring Medication Appropriateness

Data were obtained for the medication prescribed (indication, dosage and duration of treatment during the last three months or more). The presence of polymedication, defined as the regular use of five or more medications, was noted, as was that of potentially inappropriate medication (PIM) according to the STOPP v2 criteria (Screening Tool of Older Person’s Potentially Inappropriate Prescriptions, version 2) [31]. The latter variable was operationalised as the percentage of patients receiving at least one PIM.

### 2.4. Frailty Assessment

The main study outcome was frailty, assessed by the phenotype proposed by Fried et al. [5] which consists of the following criteria: (a) unintentional weight loss of 4.5 kg or more in the previous year; (b) self-reported exhaustion, identified by two questions in the Center for Epidemiological Studies Depression (CES-D) scale; (c) weakness, defined by low handgrip strength and measured in Kg in the dominant hand by using a dynamometer (Jamar hydraulic grip hand dynamometer SP-5030J1) (highest of three consecutive measurements), adjusted for gender and body mass index (grip strength was classified as low when the force exerted was below the first quintile of the distribution) (d) slow walking speed (lowest quintile of gait speed), assessed by the walking time (in seconds) over a distance of 4.57 m, adjusted for gender and height; (e) low physical activity, measured by the weighted score of kilocalories expended per week, obtained from the Minnesota Leisure Time Activity Questionnaire and adjusted for gender. Participants were classified as non-frail (robust) if they met none of the criteria, pre-frail if they met one or two criteria, and frail if three or more criteria were met.

### 2.5. Statistical Analysis

Exploratory data analysis and frequency tables were used to describe the study variables. Taking into account the three possible states of frailty (frail, pre-frail and robust), a multinomial logistic regression model was used to study the relationship between the independent variables and the outcome variable, frailty [32]. All independent variables were included in the regression model. The influence of various factors on the states of frailty and pre-frailty was examined, taking robust patients as a benchmark. Odds ratios (OR) and 95% confidence intervals (CI) were calculated for each covariate included in the model. A 5% significance level was assumed to indicate statistical significance. All statistical data analyses were performed using SPSS version 24.0 (IBM SPSS Statistics, Armonk, NY, USA).

### 2.6. Ethical Considerations

This study was conducted in accordance with the Declaration of Helsinki. The Málaga Clinical Research Ethics Committee approved the study (PI-0234-14), and informed consent was obtained from all patients prior to their inclusion.

## 3. Results

### 3.1. Characteristics of the Study Population

We included a total of 582 patients, with a mean age of 73.1 years (standard deviation 5.5, range 65–104) and slightly more than half of the sample being female. There were only eleven patients who declined to participate, which is a negligible proportion. Furthermore, as our study is based on a personal face-to-face interview in the primary care outpatient facility, there were no patients without data of our interest primary variable (frailty), and missing data were exceptional. Only 21.1% lived alone, whereas 62.4% lived with their partner (62.4%) or family (16.5%). The average CCI score was 1.48 (standard deviation 1.6, range 0–8), and 38.8% of the patients had scores greater than 2. Each patient presented 7.8 diagnoses on average (standard deviation 3.3, range 0–20). The most prevalent chronic conditions were bone and joint disorders (mainly osteoarthritis of the knee, hip, hand and shoulder) (75.3%), hypertension (70.9%) and dyslipidaemia (51.7%). Some form of psychopathology (mainly anxiety and/or depression) was present in 36% of the patients. Only 13.3% of the patients had a normal weight, while a large proportion presented overweight (40.9%) or obesity (45.7%). The mean body mass index was 30.2 (standard deviation 5.1, range 17–54.5). The mean score on the Lawton scale was 6.6 (standard deviation 1.8, range 0–8) with half of the sample being independently capable of performing IADL. Table 1 details the main characteristics of the study population. Each patient consumed on average 6.8 drugs (standard deviation 4.0; range 0–23) resulting in a polymedication prevalence pf 68.6%. Omeprazole and acetaminophen were the most prescribed drugs, followed by aspirin, simvastatin, metformin, metamizole, enalapril and bromazepam. A large proportion of patients (66.8%) presented at least one potentially inappropriate medication, according to the STOPP v2 criteria. The mean number of PIMs per patient was 2.1 (standard deviation 2.2, range 0–10). Benzodiazepines were the most frequently detected PIMs (61% of all PIMs).

### 3.2. Assessment of Frailty and Analysis of Related Factors

Among the study population of older adults, frailty was present in 24.1% (95% CI = 20.7–27.6), while 54.3% were pre-frail and 21.6% were non-frail. The most prevalent Fried phenotype criterion observed in the sample was weakness (63.9%), followed by low physical activity (48%) and exhaustion (21.3%), while unintentional weight loss (7.2%) was infrequent. These results are detailed in Table 2.

In order to further examine the impact of the independent variables on the frailty states considered, a multinomial logistic regression analysis was performed (Table 3). The main factor related to being frail was the female gender. Thus, in the sample, the female frailty odds were 20-fold the male frailty odds, all other covariates being equal. Age was also related to frailty; for each additional year of life, the odds of being frail increased by 19% (OR = 1.19, 95% CI = 1.11–1.27). The presence of frailty was also associated with the level of dependence in the IADL, with polypharmacy, with osteoarticular pathology and with the presence of mental disorder. Among the sample, the odds of frailty decreased by 47% for each additional point of independence on the Lawton scale (OR = 0.53, 95% CI = 0.42–0.67). However, the odds doubled for persons receiving polymedication (OR = 2.67, 95% CI = 1.08–6.61). The OR of people with vs. without osteoarticular pathology was 3.5 (95% CI = 1.51–8.13), and the OR of patients with vs. without psychopathology was 2.23 (95% CI = 1.12–4.44). No association was found between frailty and the other prevalent pathologies considered or with the use of at least one PIM.

The odds of an older person being pre-frail vs. robust remained unchanged concerning the association with the female gender (OR = 2.53, 95% CI = 1.52–4.23) and with age (OR = 1.09, 95% CI = 1.04–1.16).

## 4. Discussion

Overall, our results for frailty among community-dwelling older patients in Málaga (southern Spain) are consistent with those reported in similar studies conducted in other regions of Spain [33] and elsewhere in Europe [23]. The prevalence of frailty in our study population is slightly higher than the mean values reported by other studies in similar outpatient settings [34,35], and it is also higher than the mean values for Europe as a whole [21]. However, the prevalence of frailty is known to be fairly heterogeneous [19,21,25], possibly due to methodological differences that preclude direct comparison of the results obtained. The most important of these differences is the use of diverse operational definitions of frailty. Other factors that may also be relevant include the age of the persons analysed and the general characteristics of the sample (for example, the inclusion or otherwise of those who are institutionalised). In our study, the Fried frailty phenotype assessment components were used for classification because this is the most commonly used and most widely reported instrument in community settings [21]. However, the heterogeneity observed in the prevalence of frailty may also be due (at least in part) to economic and social factors or even to phenotypic diversity, i.e., expressed predominantly in the criteria associated with physical function (weakness, slowness and physical activity). In this respect, variations in the reported prevalence of frailty in Europe suggest there is a north-south gradient, producing a higher proportion of frailty and pre-frailty in Southern Europe than in northern countries [25].

Among the frailty-related criteria considered, a striking feature was the high proportion of patients who presented physical weakness, a finding consistent with the European multicentre study SHARE which reported that the prevalence of low grip strength contributed to that of frailty in Spain and Italy [25]. An earlier study of older ambulatory adults in Spain obtained results comparable to our own, namely that the most prevalent components of frailty were weakness and low physical activity [24]. According to these authors, poor muscle strength was closely associated with a lack of physical activity. In another analysis of this question, frailty has been associated with objectively-assessed sedentary behaviour patterns in older adults [36]. On the other hand, some researchers have found other criteria, such as exhaustion, to be of greater relevance [37,38]. This variability may be because the frailty syndrome does not present a single clinical course but differs according to the causes that trigger it. Differences may also arise from variations in how the dimensions of the frailty phenotype are operationalised. In our study, the original frailty phenotype criteria were applied, with the corresponding reference values, although according to some authors this approach may overestimate the prevalence of frailty [33].

In our study findings, the factor most strongly associated with frailty was the female gender, which corroborates prior longitudinal studies on ageing according to which frailty is more common among women than men, and with greater age [25]. The level of dependency for IADL was also found to be associated with frailty. According to the multinomial logistic regression model, a higher score on the Lawton scale (i.e., greater independence) significantly reduced the odds of frailty (by 47% for each additional point of independence). These data are consistent with the known relationship between frailty and disability [35,39].

With respect to comorbidities, the main variable related to frailty in our study was the presence of bone and joint disorders (especially osteoarthritis). We believe that frailty is closely linked to musculoskeletal health; indeed, musculoskeletal functioning is a key component in quantifying frailty, which is known to be associated with common age-related musculoskeletal conditions [40]. In particular, osteoarthritis, similarly to frailty, is commonly observed with increasing age. Although relatively little research has been undertaken to elucidate the mechanisms underlying the relation between osteoarthritis and frailty, potential links include elevated levels of pro-inflammatory markers in the circulatory system, age-related muscle atrophy and decreased physical activity [41]. Another factor that may provoke the development of frailty is that of chronic pain related to osteoarthritis, which could heighten the risk of physical inactivity, disability and falls [42]. The considerable prevalence of osteoarticular pathology in this sample has potentially contributed to the high frequency of the muscle weakness criterion. This criterion may therefore be overestimated with respect to populations with lower rates of said pathology, which could reduce external validity. Psychopathologies such as anxiety and/or depression may also aggravate the presence and impact of frailty among older persons. For example, a person with depressive symptoms may have a less active social life, be less physically active, consume a less healthy diet and, at the same time, be consuming psychotropic drugs. All of these consequences tend to promote or heighten frailty. In line with the above information, there are certain studies that conclude that the lack of joy and the presence of a negative attitude towards ageing can function as risk factors for frailty [43,44]. Our findings show that the presence of a psychopathology doubles the odds of an older person presenting pre-frailty. Therefore, we believe more extensive screening for depression and anxiety should be performed among older populations because these conditions tend to be under-diagnosed and because a more active approach to this question would promote healthy ageing.

Regarding medication, we observed a significant relationship between polypharmacy and frailty. This finding is consistent with previous research in which polymedication has been identified as a determinant factor of frailty [38,39,45,46,47,48,49]. The association between the two situations may be complex and bidirectional, but it seems evident that the chance of frailty developing is greater among patients who are receiving polymedication [45,47] and that the risk of a worsening transition in frail states is heightened by a greater consumption of medication [16]. Accordingly, it would seem reasonable to recommend that clinicians should seek to reduce polypharmacy in their older patients. However, although polypharmacy was associated with an increased risk of adverse events in pre-frail and frail older adults, this was not the case for non-frail individuals. Therefore, polypharmacy could be appropriate to treat multiple chronic diseases, and for robust older persons it should be managed as in younger populations [46]. Although further research is needed to confirm the possible benefits of reducing polymedication in the development, reversion or delay of frailty, it seems apparent that any treatment regimen should be evaluated with special care for frail older adults, since these patients have susceptibilities that can decrease medication benefit, as well as increase adverse secondary effects. Such an evaluation may not be straightforwardly achieved, and the identification of appropriate polypharmacy for these patients requires the development of more robust criteria for evaluating the net effects of complex medication regimens [50]. On the other hand, we found no evidence of any association between the use of one or more PIMs and the presence of frailty or pre-frailty. We speculate that such a relationship might not have been evident because the STOPP v2 criteria contain a large number of items of varying clinical significance (in terms of risk), and therefore the impact produced on frailty states by a single PIM might be slight impact. In other words, this means of measuring the risk might be insufficiently sensitive. At present, no conclusive results in this respect have been provided, except for specific medications such as anticholinergics [16,38,51] and the potentially inappropriate use of nonsteroidal anti-inflammatory drugs [52]. What does seem clear to us and to other authors [17,18] is that medication and treatment should be carefully personalised and that the regimen should be subject to periodic structured review.

The strengths of our study lie in the analysis conducted on a representative sample of healthcare centres, the global approach taken and the great variety of clinical, functional and treatment data compiled. Among its limitations is the cross-sectional design employed, which does not allow causal relationships to be established, although it can detect factors related to frailty. In addition, selecting a sample population from a single region or country may have resulted in a certain lack of external validity, even more so when taking into account the heterogeneous nature of the profile of older people living in the community. Nevertheless, the sample examined in this study is believed to be representative of the population of older adults in the ambulatory setting as long as they have a clinical and functional profiles similar to that of this sample. Furthermore, in our opinion it is preferable to assess frailty in the community as we have performed here rather than to focus more narrowly on an acute situation. A longer-term goal should be to establish frailty assessment as an integral part of routine primary care practice, as early identification and optimal management of this condition facilitates the patient’s transition towards improvement whether from a pre-frail state or when frailty is already present. This will require new prospective studies that support the evidence and possible recommendations.

## 5. Conclusions

According to the Fried phenotype, frailty may be associated with many factors, including age, female gender, dependence for IADL, polymedication, the presence of osteoarticular pathology and/or mental disorder. Some of these variables may be preventable or modifiable and, thus, provide clinically relevant targets for intervention. This is particularly the case for depressive symptoms, the clinical control of osteoarthritis and the use of polypharmacy. In this respect, many of the interventions currently being proposed are based on an effective multicomponent approach, which may include physical activity, dietary intervention, structured medication reviews and strengthened social networks.

## Figures and Tables

**Table 1:** Characteristics of the study population (*n* = 582).

Quantitative Variables	Mean	Standard Deviation
Age (years)	73.1	5.5
Lawton (IADL)	6.6	1.8
BMI (kg/m^2^)	30.2	5.1
Number of comorbidities	7.8	3.3
Number of drugs per patient	6.8	4.0
Number of PIMs per patient	2.1	2.2
**Qualitative Variables**	**Subjects (*n*)**	**Percentage (%)**
Gender Male Female	248 334	42.6 57.4
Lawton (IADL) 0–1 2–3 4–5 6–7 8	12 37 72 165 295	2.1 6.4 12.4 28.4 50.8
SPMSQ (Pfeiffer) 0–2 errors 3–4 errors 5 errors and over	533 36 11	91.9 6.2 1.9
GDS-15 0–5 6–9 10 and over	440 107 33	75.9 18.4 5.7
BMI categories Underweight Normal Overweight Obese	1 77 237 265	0.2 13.3 40.8 45.7
Nutritional status Normal Malnutrition risk Malnourished	552 20 7	95.3 3.5 1.2
Charlson Comorbidity Index 0–1 2 3 and over	356 106 120	61.2 18.2 20.6
Most frequent comorbidities Bone and joint disorders Hypertension Dyslipidaemia Insomnia Gastrointestinal disease Psychopathology Diabetes mellitus Heart disease Respiratory disease	438 412 301 258 249 210 176 169 125	75.3 70.9 51.7 44.3 42.4 36.1 30.3 29.1 21.5
Polymedication	399	68.6
PIM prevalence	389	66.8

IADL: Instrumental activities of daily living; BMI: Body mass index; PIM: Potentially inappropriate medication (according to STOPP v2 criteria); SPMSQ: Short Portable Mental Status Questionnaire (0–2 errors: normal mental functioning; 3–4 errors: mild cognitive impairment; 5 errors and over: moderate-severe cognitive impairment); GDS-15: Geriatric Depression Scale (0–5: no depression; 6–9 suggestive of depression; 10 and over: almost always depression).

**Table 2:** Frailty states and criteria according to Fried’s phenotype (*n* = 582).

	Subjects (*n*)	Percentage (%)
**Frailty states** Robust (non-frail) Pre-frail Frail	126 316 140	21.6 54.3 24.1
**Fried criterion** Unintentional weight loss Exhaustion Weakness Slow walking speed Low physical ctivity	42 124 372 113 279	7.2 21.3 63.9 19.4 48.0

Robust: 0 criteria present; Pre-frail: 1–2 criteria present; Frail: 3 or more criteria present.

**Table 3:** Factors related to frailty. Multinomial logistic regression for frail and pre-frail states (with respect to non-frail).

Independent Variable	Frail OR (95% CI)	Pre-Frail OR (95% CI)
**Age**	1.19 (1.11–1.27) ***	1.09 (1.04–1.16) **
**Charlson comorbidity index**	1.27 (0.99–1.62)	1.08 (0.85–1.31)
**Lawton-Brody (IADL)**	0.53 (0.42–0.67) ***	0.97 (0.81–1.17)
**Gender** Female Male	20.54 (9.10–46.3) *** 1	2.53 (1.52–4.23) *** 1
**Diabetes mellitus** Yes No	1.82 (0.83–3.96) 1	1.09 (0.61–1.98) 1
**Heart disease** Yes No	1.74 (0.82–3.69) 1	1.15 (0.64–2.08) 1
**Respiratory disease** Yes No	1.47 (0.50–2.85) 1	2.17 (1.14–4.10) * 1
**Bone and joint disorder** Yes No	3.51 (1.51–8.13) ** 1	1.36 (0.83–2.21) 1
**Psychopathology** Yes No	2.23 (1.12- 4.44) * 1	2.01 (1.17–3.46) * 1
**Polypharmacy** Yes No	2.67 (1.08–6.61) * 1	0.94 (0.55–1.61) 1
**PIM** At least one None	2.95 (0.66–3.13) 1	0.77 (0.46–1.26) 1

OR: odds ratio; 95% CI: 95% Confidence Interval; IADL: Instrumental activities of daily living (numerical value of the Lawton Index); PIM: Potentially inappropriate medications (according to STOPP v2 criteria). * *p* < 0.05; ** *p* < 0.01; *** *p* < 0.001.

## Data Availability

The data that support the findings of this study are not publicly available due to being used for further investigational objectives and because the data contain information that could compromise the privacy of research participants. However, specific information can be obtained from the corresponding author upon reasonable request (E.B.-R.).
